# 

**Published:** 1920-10-09

**Authors:** 


### THE HOSPITAL

**The Modern Newspaper of Administrative Medicine and Institutional Life.**

Telegraphic Address: OSPEDALE, RAND, LONDON. Telephone Number: 2734 GERRARD.

No. 1792, Vol. LXIX. | Saturday, October 9th, 1920. | PRICE TWOPENCE.

